# A SAR and optical remote sensing dataset in Seville for scientific research in agriculture

**DOI:** 10.1038/s41597-026-07419-5

**Published:** 2026-05-08

**Authors:** Arturo Villarroya-Carpio, Víctor Cazcarra-Bes, Alejandro Mestre-Quereda, Juan M. Lopez-Sanchez

**Affiliations:** 1 Institute for Computer Research, University of Alicante, Alicante, Spain; 2 Department of Geoscience and Remote Sensing, Faculty of Civil Engineering and Geosciences, TU Delft, Delft, The Netherlands

## Abstract

Radar remote sensing data is underused for agricultural applications due to the complexity of its pre-processing and to the non-obvious physical interpretation of the derived features. To address these challenges, this work presents the SAR and Optical Dataset for Agriculture in Seville (SODAS), which integrates time series of radar images (Sentinel-1), optical images (Sentinel-2), precipitation records, and crop-type maps. The georeferenced images cover an agricultural area in Seville, Spain, from 2017 to 2021. The SAR images are provided in the form of dual-polarimetric covariance matrices, which include the backscattering coefficient and the correlation between channels, and repeat-pass interferometric products (coherence and phase) at VV and VH polarimetric channels. The optical images correspond to reflectivity at red, green, blue, and near infra-red bands, as well as NDVI products. This dataset has many potential uses, such as development of algorithms for crop-type mapping, retrieval of biophysical parameters, crop monitoring, and data fusion. Additionally, a Jupyter notebook to load the dataset, create and compare time series, and visualise images is included.

## Background & Summary

The use of remote sensing data is well established in the context of agricultural monitoring, as an automatic and non-destructive way to get information about vegetation on local to global scale^1^. Optical remote sensing has been the main tool used for multiple purposes related to agriculture over the last decades because optical data are characterised by a clear interpretation and good ability to describe crop physiological phenomena. Applications include, among others, crop type classification^2,3^, crop monitoring and health assessment^4^, yield estimation^4–6^, retrieval of biophysical variables^7,8^, soil moisture and irrigation management^4,9,10^, and precision farming^11,12^.

However, the use of optical imagery is limited by the atmospheric conditions. Although atmospherical effects can be taken in consideration, clouds may completely block the scene. In addition, the images have to be acquired during daytime. Contrarily, radar images provide an improved temporal coverage, not being affected by clouds or the day-light requirement. Consequently, synthetic aperture radar (SAR) data have been successfully used in multiple applications related to agriculture^13–15^.

In the past, most studies exploiting SAR data in agriculture applications were based on the backscattering coefficient as the main input product, which corresponds to the intensity of the images. However, SAR sensors provide images that are not limited to intensity or gray-level values, but incorporate additional features which can be exploited in many applications because they offer complementary sensitivity to different aspects of the scene. These additional features are polarimetry and interferometry.

Polarimetry denotes the study of the change induced by the scene on the polarisation of the waves, for which different combinations of transmitted and received polarisations are measured^16,17^. Polarimetry is sensitive to the shape, orientation, size, and dielectric properties of the elements present in the scene, hence offering rich information in areas with crops^15,18^. Regarding radar interferometry, defined as a coherent combination of two radar images^19,20^, it provides sensitivity to the vertical structure of the scene and/or to changes produced between the two images. For instance, repeat-pass interferometric coherence has been evaluated for crop monitoring^21–23^ and crop classification^24^, whereas single-pass coherence is suited for crop height retrieval^25,26^.

Unfortunately, the use of SAR data requires more complex processing than optical data, and the interpretation of the radar-derived features is not straightforward in many occasions. Consequently, SAR data are largely underused in agricultural applications.

After the launch of Sentinel-1 in 2014, which offers a revisit cycle of 6 or 12 days, depending on the year and the geographical location, and an open-access data policy, the adoption of SAR data in agriculture studies has increased. For this purpose, users mainly rely on cloud-based services, such as Google Earth Engine, which provide pre-processed Sentinel-1 images in analysis ready data (ARD) format and a suite of geospatial analysis tools. However, the pre-processed images only include the backscattering coefficient (intensity) of the images at the two polarimetric channels (VV and VH), hence strongly limiting the observation space with respect to the potential use of polarimetry and interferometry. The dataset described in this manuscript is aimed at offering all observation dimensions available in Sentinel-1 data, hence covering intensity, polarimetry and interferometry, in an ARD format. Its final purpose is providing the opportunity to non-experts in radar to explore the potential of this type of data avoiding the limitations (i.e. intensity only) of current cloud-based services and without requiring any further processing.

The dataset consists of a uniform time series of ARD products, spanning five years, acquired over the same agricultural area, and comprising geocoded dual-pol covariance matrix and geocoded repeat-pass complex coherence.

Parts of this dataset were used in the past in four publications. Di Martino *et al*.^27^ exploited a subset formed by backscatter and coherence time series over 2017 to analyse the performance of autoencoders to describe the temporal features of each crop type, which led to the detection of mislabelled fields. A specific application for mislabel detection was devised later by the same authors using the same year and radar features^28^. In parallel, Villarroya *et al*.^23^ studied the use of the repeat-pass interferometric coherence as a vegetation index by comparing it with NDVI using the data from 2017. The processing requirements and the influence of orbit was assessed by comparing three different orbits. That study on the coherence exploitation as a vegetation index was later extended in time by including all five years considered in this dataset (2017–2021)^29^.

Regarding other datasets relevant to this field, there are some specific datasets that have been made publicly available recently which include repeat-pass interferometric coherence from Sentinel-1. For instance, a global dataset of Sentinel-1 backscatter and repeat-pass interferometric coherence for all the land masses and ice sheets from 82^∘^ Northern to 79^∘^ Southern latitudes^30^. A multi-year dataset of coherence, spatially averaged per crop field, covering all agricultural areas in The Netherlands was also published^31^.

In summary, this article describes a new dataset containing both optical and radar data over croplands, designed to facilitate the exploration of their information content and the comparison between both types of data in agricultural applications such as growth monitoring and crop-type mapping. This dataset is especially suited for assessing the use for agriculture of both polarimetric and interferometric features derived from Sentinel-1.

It is important to emphasise that, while amplitude-only radar products are broadly available, e.g. on cloud platforms, interferometric coherence (complex coherence magnitude and phase) and polarimetric covariance matrix are far less common in public datasets, mainly because their generation requires non-trivial processing. By providing a long time series of advanced products already computed, this dataset enables users, especially non-SAR specialists, to explore and exploit interferometric and polarimetric information without needing a dedicated processing pipeline or expertise. The use of a common grid for all the different products included simplifies the post-processing, providing a complete dataset ready to be analysed. The availability of SAR, optical, and reference data in the same dataset makes the dataset workflow-ready, i.e. a user can easily work with the data without a deep expertise on remote sensing. In addition, simple scripts are provided to read and use the data, which makes it suitable not only for remote sensing experts but also for data scientists and for educational use in courses where the focus is on interpretation and modelling rather than on data wrangling and SAR processing.

## Methods

This section is divided into two subsections. The first subsection describes the input data employed to build the dataset: crop inventories, Sentinel-1 images, and Sentinel-2 images. The second subsection is devoted to explain the processing carried out with all input data to generate the dataset.

### Input data

#### Crop-type reference data

The reference data used to create the crop-type maps included in the dataset were provided by the Regional Government of Andalusia (*Consejería de Agricultura, Pesca, Agua y Desarrollo Rural, Junta de Andalucía*). The original data to generate the crop-type maps can be accessed under request in a dedicated portal^32^. The input reference dataset was provided in vector form and consisted of five SHP files, one for each year from 2017 to 2021, covering the whole province of Seville, Spain. The vector files contain, as one of the data fields, the official declarations of the farmers, which indicate the type of crop cultivated within each polygon at each year.

**Fig. 1:**
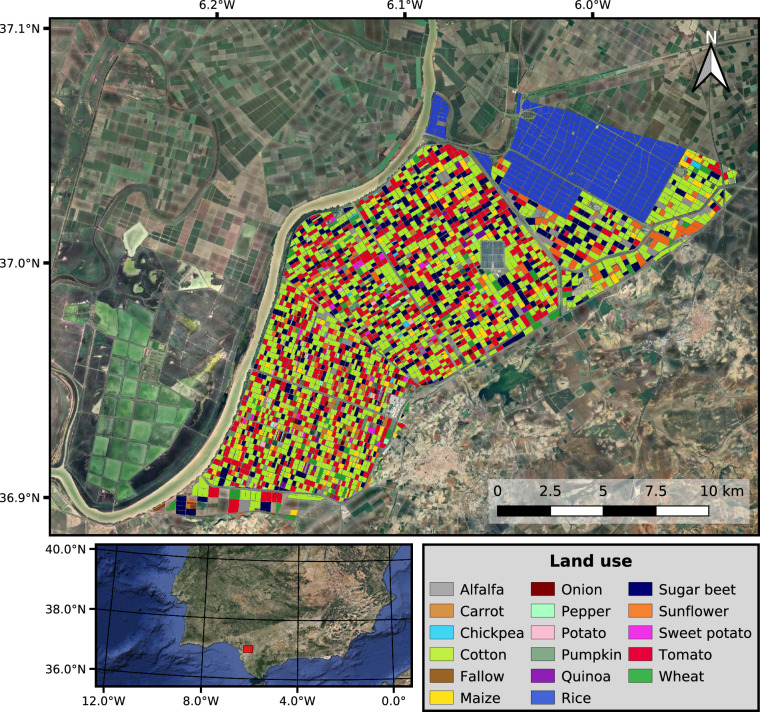
Location of the study site in Seville (Spain), showing the reference data with the main crops cultivated in 2017 (©Google Earth 2017).

A spatial subset of around 30 × 30 km was extracted for further processing, covering an agricultural area centred at 37 N 6.1 E (Fig. 1). In this region, a wide variety of crops are planted, with rice and cotton being predominant. Eighteen crop types occupy the largest surface (between 94% and 98% depending on the year) and hence they were selected as the classes used to create the crop-type maps employed in the dataset. The area covered each year by every crop type is detailed in Table 1. The same crop types and years were used in a previous study, so the same values were reported in Villarroya *et al*.^29^.

**Table 1:** Total area (km^2^) occupied by each crop every year.

Crop Type	2017	2018	2019	2020	2021
Alfalfa	4.10	3.26	2.14	2.42	3.05
Barley	—	1.81	1.05	0.73	1.06
Carrot	2.22	0.94	1.06	1.82	2.24
Chickpea	0.55	1.02	0.48	1.11	0.61
Cotton	60.47	82.10	78.16	77.69	80.54
Fallow	1.17	4.68	3.36	4.39	19.82
Maize	6.73	5.59	10.81	4.00	4.07
Oats	—	1.69	1.29	0.87	0.18
Onion	1.58	2.25	3.66	6.22	5.36
Pepper	0.67	3.59	4.16	4.68	3.80
Potato	0.43	0.50	0.37	0.88	—
Pumpkin	0.96	1.18	1.17	1.01	0.87
Quinoa	1.08	0.74	1.53	2.31	2.63
Rice	27.81	29.62	30.35	31.19	17.46
Sugar beet	21.23	19.48	14.42	14.81	12.87
Sunflower	6.31	4.79	3.16	4.13	4.32
Sweet potato	1.27	2.04	3.14	2.38	0.56
Tomato	32.46	27.96	35.45	32.88	34.76
Wheat	4.38	4.87	3.29	4.49	2.80
TOTAL	173.43	198.11	199.05	198.02	196.99

Precise information about the growing period (e.g. planting and harvest dates) of each individual field, gathered by in situ surveys, is not provided by any institution in this region. Readers interested in using approximate values of the growing period for each year can access the corresponding information provided by the Copernicus Land Monitoring Service (CLMS, https://land.copernicus.eu/en), within the set of Bio-geophysical variables, Vegetation subset, products named *Start-of-season Date* and *End-of-season Date*. These products indicate the months of start and end of growing season and are derived from time series of optical images of Sentinel-2. As they are derived externally to this research, they are not included in the dataset.

#### Sentinel-1 images

All the Sentinel-1 SLC products covering the test site from orbit 74 during the 2017–2021 period were selected. All Sentinel-1 images were downloaded from the Alaska SAR Facility (https://search.asf.alaska.edu). For this specific orbit, the images are acquired in ascending mode at around 6 p.m., and the acquisition angle over the study area ranges between 31.5° and 33.5°.

#### Sentinel-2 images

All the available reflectance images of Sentinel-2 from orbit 137 (acquired between 11:00 and 11:20) with Level-2 A processing were pre-selected. The products were downloaded from the French Theia Land Data Centre (https://www.theia-land.fr/en/homepage-en) in TIFF format.

#### Precipitation data

Daily rainfall data were obtained from the Agroclimatic Information System for Irrigation (SIAR) of the Government of Spain (Ministerio de Agricultura, Pesca y Alimentación), and downloaded at the following website: https://servicio.mapa.gob.es/siarweb. They correspond to the Lebrija I station, located in the centre of the study site.

### Data processing

#### Satellite data

**Fig. 2:**
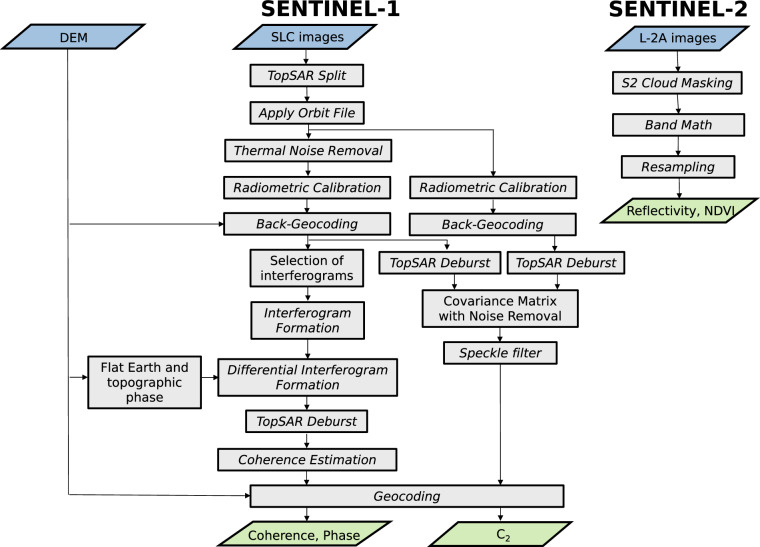
Processing flowchart for the satellite images.

The processing flowchart applied to the series of satellite images is shown in Fig. 2. The processing was carried out using ESA SNAP software, for which dedicated python scripts were implemented to carry out all steps in batch mode. For the processing of Sentinel-1 images, the ESA SNAP software automatically downloads, from dedicated servers, the precise orbit files and the required tiles of a digital elevation model (DEM). The SRTM DEM was chosen, but the study area is very flat and, hence, any DEM would provide good results for the generation of this dataset.

The processing carried out with the Sentinel-1 images comprises all the steps required to form interferometric products (complex coherence) and polarimetric products (covariance matrix). After radiometric calibration, all the Sentinel-1 images were co-registered with respect to a reference image using the *back-geocoding* algorithm. Images were processed with and without passing through the *thermal noise removal* step because both types of data are required for a noise removal algorithm especially designed for the dual-pol covariance matrix^33^, which was then applied. The covariance matrices were estimated using a boxcar filter with kernel size 4 × 19 (azimuth × range). In parallel, starting from the co-registered images, all interferograms with temporal baselines of 6 and 12 days were computed and the phase components related to flat Earth and topography were removed, hence producing differential interferograms. The complex coherence was estimated using also a boxcar filter with the same size as the polarimetric covariance matrix. Finally, both the interferometric coherence (magnitude and phase) and the entries of the dual-pol covariance matrix were geocoded to an output grid in UTM coordinates with 10 m posting.

As for the optical data from Sentinel-2, the images were masked using the cloud mask layer provided with the product. Images with a proportion of clouds above 75% were discarded and removed from the dataset. A mosaicking was performed to combine the two adjacent tiles covering the area (29SQA and 29SQB). Then, the NDVI images were constructed using the *band math* tool from SNAP. Finally, the layers were resampled to the same grid used by the Sentinel-1 images by means of GDAL routines^34^.

#### Crop-type maps

For each one of the five years, the spatial subset of the vector layer with the crop-type data which coincided with the same extent of the satellite products was extracted and rasterized with a 5 m posting. A morphological erosion was applied to the layer, removing a 5 m buffer from the border of all polygons. Subsequently, the layer was resampled to the same grid used in the satellite products. Finally, a code 0 (meaning *No crop*) was assigned to all pixels belonging to any crop class occupying a total surface inferior to 0.1 km^2^.

#### Precipitation data

The daily precipitation data were included in the dataset without any processing.

## Data Records

**Fig. 3:**
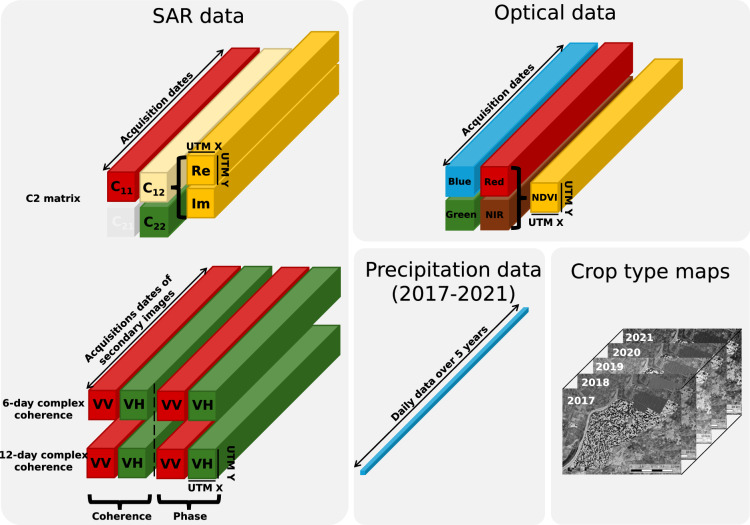
Internal structure of the image time series stored in the NetCDF file. The crop-type maps are represented over a grayscale optical image (©Google Earth 2017).

The dataset^35^ is stored in a single NetCDF-5 (Network Common Data Form) file. Its internal structure, illustrated in Table 2 and Fig. 3, is as follows:

- Time series of SAR data over the years 2017 to 2021, in the form of the elements of the *C*_2_ matrix: *C*_11_, real(*C*_12_), imag(*C*_12_), and *C*_22_. Each year includes 60-61 acquisitions with a 6-day revisit time, except for 2021, due to the failure of Sentinel-1B on December 23. The result is an array, “S1_C2”, of dimensions 301 × 4 × 3588 × 3607, corresponding to the number of images (Table 3), the real components of the *C*_2_ matrix, and the dimensions of the georeferenced images.
- Time series of 6-day repeat-pass coherence at VV and VH polarisations. The date of each interferometric product is defined as the date of the second (most recent) acquisition. Coherence and phase are stored separately (“S1_06days_coherence” and “S1_06days_phase”) and both share the same shape: 298 × 2 × 3588 × 3607.
- Analogous time series for a 12-day temporal baseline. The 12-day interferograms were constructed using all acquisitions, resulting in alternating pairs of images from Sentinel-1 A and Sentinel-1 B.
- Time series of S2 images, including bands 2, 3, 4 and 8, corresponding to blue, green, red, and near infrared (NIR) respectively; and the NDVI. The series are all stored in an array of shape 210 × 5 × 3588 × 3607, following the same logic as with the S1 imagery.
- A set of 5 crop-type maps (one per year) in the form of a raster with integer values ranging from 0 to 19. The code correspondence is shown in Table 4
- A time series of daily precipitation (mm/day) data covering the period between 2017 and 2021.

**Table 2:** Internal structure of the image time series stored in the NetCDF file. Series within the same black box are stored as a single variable in the file.

Variable	Dimensions	Size	Description
S1_C2	(time C2, element C2, y, x)	(301, 4, 3587, 3607)	Sentinel-1 covariance matrix elements
S1_06days_coherence	(time coher 06days, channel Cband, y, x)	(298, 2, 3587, 3607)	Sentinel-1 6-day coherence magnitude
S1_06days_phase	(time coher 06days, channel Cband, y, x)	(298, 2, 3587, 3607)	Sentinel-1 6-day interferometric phase
S1_12days_coherence	(time coher 12days, channel Cband, y, x)	(297, 2, 3587, 3607)	Sentinel-1 12-day coherence magnitude
S1_12days_phase	(time coher 12days, channel Cband, y, x)	(297, 2, 3587, 3607)	Sentinel-1 12-day interferometric phase
S2	(time optical, band optical, y, x)	(210, 5, 3587, 3607)	Sentinel-2 optical reflectance & NDVI
crop_type_map	(year, y, x)	(5, 3587, 3607)	Annual crop type map
precipitation	(time precipitation,)	(1826,)	Daily accumulated precipitation
times_C2	(time C2,)	(301,)	Timestamps for S1 C2
times_coher_06days	(time coher 06days,)	(298,)	Timestamps for 6-day coherence/phase
times_coher_12days	(time coher 12days,)	(297,)	Timestamps for 12-day coherence/phase
times_optical	(time optical,)	(210,)	Timestamps for optical data
times_precipitation	(time precipitation,)	(1826,)	Timestamps for precipitation data
x	(x,)	(3607,)	Easting coordinates(m)
y	(y,)	(3587,)	Northing coordinates (m)
elements_C2	(element C2,)	(4,)	Elements of covariance matrix
channels_Cband	(channel Cband,)	(2,)	VV and VH channels
bands_optical	(band optical,)	(5,)	Optical bands
crops	(crop,)	(19,)	Crop classes
years	(year,)	(5,)	Crop map years
crop_type_dictionary	(crop,)	(19,)	Mapping from crop ID to crop label

**Table 3:** Number of Sentinel-1 and Sentinel-2 products per year.

Year	2017	2018	2019	2020	2021
*C*_2_ matrix	61	60	61	61	58
6-day complex coherence	60	59	61	61	57
12-day complex coherence	59	59	61	61	57
Optical bands and NDVI	39	45	47	40	39

**Table 4:** Code assigned to each of the crops included in the crop class maps.

Code	Crop	Code	Crop	Code	Crop
0	**No crop**	7	Maize	14	Rice
1	Alfalfa	8	Oats	15	Sugarbeet
2	Barley	9	Onion	16	Sunflower
3	Carrot	10	Pepper	17	Sweet potato
4	Chickpea	11	Potato	18	Tomato
5	Cotton	12	Pumpkin	19	Wheat
6	Fallow	13	Quinoa		

## Technical Validation

The technical quality of the dataset relies on three aspects: the sources of the data, the processing carried out, and a set of sanity checks on the final product.

### Data sources

The main contents of the dataset are the Sentinel-1 and Sentinel-2 images. The quality of the images provided by these satellite constellations is constantly checked and verified as part of mission performance controls carried out by the European Space Agency (ESA)^36^. The SAR Mission Performance Cluster (SAR-MPC) for Sentinel-1, and the Optical Mission Performance Cluster (OMPC) for Sentinel-2, are in charge of the End-To-End instrument and product performances for these Copernicus missions. This includes the operation of the service with daily quality control of the products, the calibration and validation of the products, and the maintenance of the processors. All product quality metrics relevant to the images are evaluated and reported periodically by the SAR-MPC and the OMPC.

As for the annual crop-type reference maps, they are derived from the official Land Parcel Identification System (LPIS)^37^, called SIGPAC in Spain, which is a geographical information system that provides a graphical declaration of land parcels for farmers. This system is a key component for the European Union’s Common Agricultural Policy (CAP) subsidy schemes. It integrates both graphic (parcels as polygons) and alphanumeric data to enable administrative checks and on-the-spot inspections by authorities to ensure eligibility for aid and subsidies. As a public and administrative record of all agricultural land, it is frequently checked and scrutinized by the administration.

Finally, the daily precipitation data comes from a standard automated meteorological station operated by the Government of Spain. Before publication, all data follow the validation procedure described in the UNE 500540:2004 standard for *Automatic weather stations networks: Guidance for the validation of the weather data from the station networks and real time validation*.

### Processing

Both the Sentinel-1 and Sentinel-2 images were processed with ESA SNAP V10.0 software^38^, which is considered as a reference tool for the processing of the images of all Copernicus missions and, hence, ensure the quality of the processing. As an example, a similar publication of Sentinel-1 data in this journal has proven the quality of SNAP as a state-of-the-art processing tool for Sentinel-1 data^31^.

The rasterization and code conversion of the crop-type information into rasters with the same grid as the satellite images maps were performed using GDAL routines, which are also a reference library in geospatial data processing.

The precipitation data were not processed.

### Sanity checks

Finally, once the dataset was formatted as a single file, the following checks were carried out to ensure the technical quality of the data.

- The accuracy of the orthorectification (or geocoding) of Sentinel-1 images carried out by SNAP is known to be below the final pixel size (or posting) of the products included in the dataset, which is 10 m.
- As an additional quality check, the geocoding of the reference image was checked by qualitatively inspecting the position of 20 representative elements (cross-roads, corners of fields, etc.) distributed all over the study site by overlaying it to optical images. The rest of the Sentinel-1 SLC images were accurately coregistered respect to the reference image, hence they share the same geocoding accuracy.
- All raster products derived from satellite data and from the crop-type data were checked to present the same size and geographical metadata.
- The data values were further checked to present values in the expected range or interval, e.g. NDVI between −1 and 1, coherence amplitude between 0 and 1, interferometric phase between  − *π* and *π*, crop-type codes in the same set shown in Table 4, precipitation positive or zero, etc.
- For the Sentinel-1 constellation, the spatial baseline is very small since the control of the orbital tube is very precise. More precisely, as indicated in the reference documents of the mission^36^, the orbital tube has a deviation of  ±120 m. This leads to a small perpendicular baseline between acquisitions, which translates into a very small decorrelation of the coherence due to the baseline length.

**Fig. 4:**
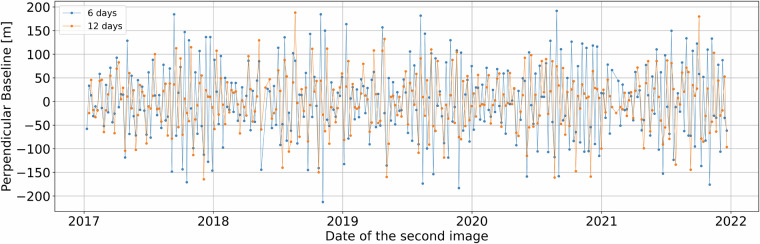
Perpendicular baseline of each interferometric pair. Both 6-day and 12-day pairs are shown.

To confirm the expected values, Fig. 4 presents the perpendicular baseline for the 6- and 12-day cases. As shown in the figure, only a small number of baselines are larger than +/- 150 meters. For the complete time series, the mean absolute values are 66.2 m and 48.3 m for the 6-day and 12-day interferometric pairs, respectively. With these values, and considering that the critical baseline is around 5 km, a negligible baseline-induced decorrelation compared to temporal decorrelation due to scene features is expected.

## Usage Notes

This dataset allows for a wide analysis of the spatial and temporal characteristics of both polarimetric and interferometric features derived from Sentinel-1 data over a broad variety of crop types and during five whole years. Since all Sentinel-1 data are geocoded and provided in the same geographical grid as the crop-type maps and the Sentinel-2 images, an exhaustive study on the correlation among different observations and features can be carried out. The precipitation data provided can also help study the influence of rain over the different observables. Different types of models, from physically-based to machine learning approaches, can be used to explore the relationship among diverse variables and the sensitivity that each one provides to the growth of crops.

**Fig. 5:**
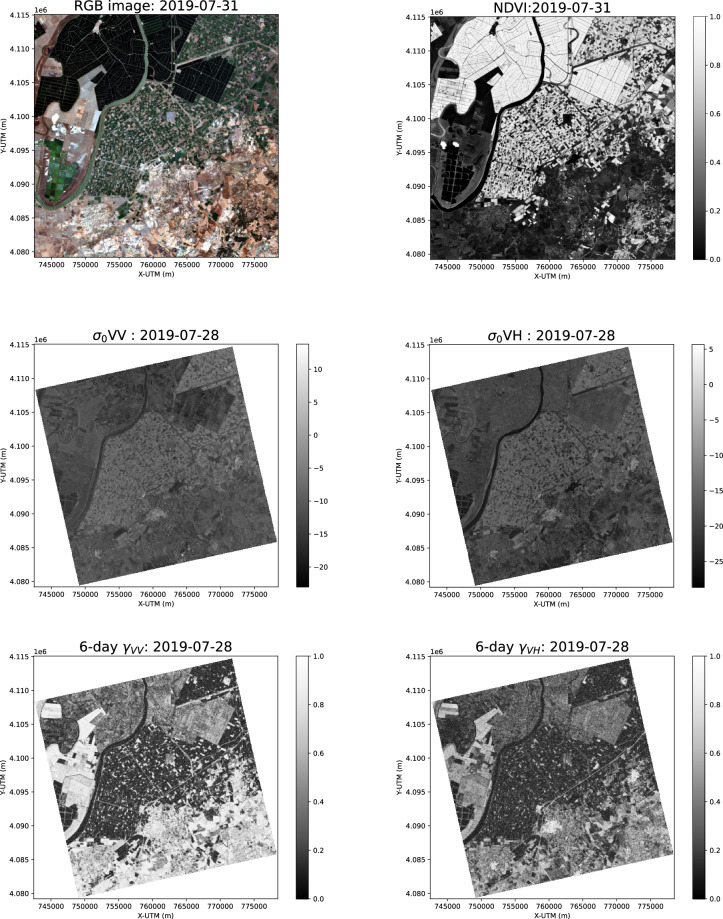
Examples of Sentinel-1 and Sentinel-2 images at the end of July 2019. From top to bottom: RGB and NDVI images from Sentinel-2, *σ*_0_ from Sentinel-1 at both channels, and 6-day coherence amplitude from Sentinel-1 at both channels.

Figure 5 shows some examples of the Sentinel-1 and Sentinel-2 images included in the dataset. The images are acquired in July 2019, when the summer crops are near their peak growth stage. This is reflected in the RGB image, where a majority of fields are shown in green, as well as in the NDVI image, which shows very high values in the central part of the site and in the rice fields in the northern part of the image. A similar behaviour is seen in the backscatter (the images in the second row), where the intensity is generally higher over vegetated areas. The opposite is true in the case of coherence. In this case, if the scene is stable over time, coherence is generally high, as temporal decorrelation is low. In the presence of vegetation, changes induced by plant growth, by fluctuations in vegetation water content, and by movement caused by wind, produce a decrease in coherence.

**Fig. 6:**
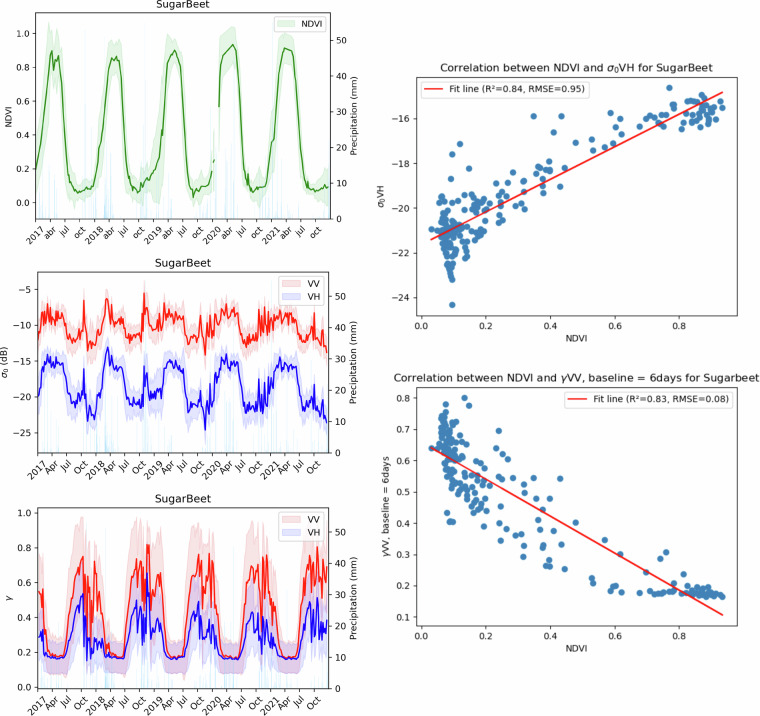
Left column: time series of NDVI (top), backscattering coefficient (centre) and 6-day interferometric coherence (bottom) for sugar beet during the whole period (2017–21). The plots show the average values for all sugar beet fields on each date. The shaded areas represent +/−1 standard deviation, and the light blue bars represent the daily precipitation. Right column: scatter plots comparing NDVI with backscattering coefficient at VH channel (top) and 6-day coherence at VV channel (bottom). The average value at each date is employed in the scatter plots.

Apart from the images, one can easily build time series to measure the evolution of different Earth Observation (EO) features over specific crop types. For instance, Fig. 6 presents the whole time series (5 years) of NDVI, backscattering coefficient and 6-day interferometric coherence for sugar beet. As mentioned before, the high values of NDVI, corresponding to each growing season, are matched by increased values of the backscattering coefficient, which are more pronounced in the VH channel. However, there are also some peaks of backscatter due to rain events because precipitation causes an increase in both soil moisture and vegetation surface water content. This makes the backscatter series noisier than NDVI, especially in the dates out of the growing season. Coherence shows a behaviour opposite to backscatter, with low values during the growing season and higher values in the out-of-season periods. Coherence at the VV channel exhibits a larger dynamic range than at the VH channel. The effect of rain events on the coherence time series consists in marked dips of coherence in the out-of-season periods, as rain generates a strong temporal decorrelation.

The time series can be exploited to measure the correlation between different EO products. For instance, the scatter plots shown in Fig. 6 compare the time series of NDVI with the time series of coherence at VV channel and backscattering coefficient at VH channel, for sugar beet.

## Data Availability

The SODAS dataset, as well as the supplementary code, are publicly available in the Zenodo repository. Version 1.1 is described in this publication and can be accessed at 10.5281/zenodo.15520299. In case of updates, go to 10.5281/zenodo.15342790 for the latest version.

## Code availability

Two supplementary python files are included along with the dataset:

• SODAS_dataset_inspection.ipynb

• functions.py

The first one is a Jupyter notebook designed to explore and illustrate the internal structure of the dataset, as well as to provide examples about how to query specific data (products, dates, channels, etc.), visualise images of the different data types, generate time series, and analyse correlations between them. The auxiliary functions required in this notebook are all defined in the functions.py file.

The python packages required to run the supplementary scripts are:

• numpy

• matplotlib

• scipy

• sklearn

• netCDF4
